# AGT haplotype in *ITGA4* gene is related to antibody-mediated rejection in heart transplant patients

**DOI:** 10.1371/journal.pone.0219345

**Published:** 2019-07-23

**Authors:** Lucía Núñez, Grecia M. Marrón-Liñares, María G. Crespo-Leiro, Eduardo Barge-Caballero, Eloy Álvarez-López, Natalia Suarez-Fuentetaja, María Jesús Paniagua-Martin, Jorge Pombo, Javier Muñiz, Carmela D. Tan, E. René Rodríguez, José Manuel Vázquez-Rodríguez, Manuel Hermida-Prieto

**Affiliations:** 1 Grupo de Investigación en Cardiología, Instituto de Investigación Biomédica de A Coruña (INIBIC), Complexo Hospitalario Universitario de A Coruña (CHUAC), Sergas, Universidade da Coruña (UDC), A Coruña, Spain; 2 Servicio de Cardiología, Instituto de Investigación Biomédica de A Coruña (INIBIC), Complexo Hospitalario Universitario de A Coruña (CHUAC)-CIBERCV, Sergas Universidade da Coruña (UDC), A Coruña, Spain; 3 Servicio de Anatomía Patológica, Instituto de Investigación Biomédica de A Coruña (INIBIC), Complexo Hospitalario Universitario de A Coruña (CHUAC), SERGAS, A Coruña, Spain; 4 Department of Pathology, The Cleveland Clinic, Cleveland, Ohio, United States of America; University of Toledo, UNITED STATES

## Abstract

**Introduction:**

One of the main problems involved in heart transplantation (HT) is antibody-mediated rejection (AMR). Many aspects of AMR are still unresolved, including its etiology, diagnosis and treatment. In this project, we hypothesize that variants in genes involved in B-cell biology in HT patients can yield diagnostic and prognostic information about AMR.

**Methods:**

Genetic variants in 61 genes related to B-cell biology were analyzed by next generation sequencing in 46 HT patients, 23 with and 23 without AMR.

**Results:**

We identified 3 single nucleotide polymorphisms in *ITGA4* gene (c.1845G>A, c.2633A>G, and c.2883C>T) that conformed the haplotype AGT-ITGA4. This haplotype is associated with the development of AMR. Moreover, AMR patients with the haplotype AGT-ITGA4 present lower levels of integrin α-4 in serum samples compared to the reference GAC haplotype in control patients.

**Conclusion:**

We can conclude that polymorphisms in genes related to the biology of B-cells could have an important role in the development of AMR. In fact, the AGT haplotype in *ITGA4* gene could potentially increase the risk of AMR.

## Introduction

The spectrum of clinical rejection in heart transplantation (HT) involves both arms of the adaptive immune response, the T cell-mediated response leading to cellular rejection (CR), and the humoral response leading to antibody-mediated rejection (AMR) [1]. The humoral arm of the immune response is dominated by B-cells and production of antibodies. Administration of immunosuppressive agents enables more than 90% of heart transplant patients to survive longer than a year. However, conventional immunosuppressive agents are more effective in preventing responses by T cells than responses by B-cells and, therefore, conventional therapies have little impact on AMR [2]. Thus, the treatment of clinical and subclinical AMR remains sub-optimal and new approaches are needed to improve it.

The occurrence and severity of AMR are variable, and genetic polymorphisms that affect the magnitude and nature of the B-cell response are likely to contribute to such phenotypic variation. In fact, several studies investigated the relationship between single nucleotide polymorphisms (SNPs) in genes known to impact B-cell activation/function and antibody effector function [3,4]. In the field of transplantation, most of these studies were performed in kidney transplantation and the strongest evidence available suggests that variants affecting the expression of immunomodulatory cytokines, particularly IL-10, may impact on renal transplant outcomes [4–7]. To our knowledge, no study has thoroughly examined the relationship of variants in genes related to B-cell biology and AMR in HT.

We hypothesize that variants in exon-coding sequences and surrounding area of genes involved in B-cell biology in HT patients can yield diagnostic and prognostic information about AMR. Thus, identifying variants associated with AMR might allow risk stratification of patients and a genetic-based immunosuppression regimen.

## Materials and methods

### Study design and patients characteristics

This is a retrospective case-control study to evaluate a set of 61 genes related to the biology of B-cells (S1 Fig, S1 Table) and its association with AMR in HT patients from the Advanced Heart Failure and Transplant Unit of the *Complejo Hospitalario Universitario de A Coruña* (CHUAC). The study was carried out on 46 HT recipients, 23 with and 23 without AMR diagnosis, who underwent the transplantation between June 2000 and November 2016 [8]. Due to the different AMR criteria in the last years, in this study the patients before 2013 (n = 15) were diagnosed as: (1) allograft dysfunction (left ventricular ejection fraction <30% and/or heart failure), (2) no evidence of other causes of allograft dysfunction (acute cellular rejection or CAV), (3) evidence of complement activation on endomyocardial biopsy (C4d and/or C3d staining), and (4) favourable response to therapy addressing AMR (e.g., plasmapheresis, rituximab, steroid boluses). Whereas AMR in patients who underwent HT after 2013 (n = 8) was classified according to International Society for Heart and Lung Transplantation (ISHLT) [9]. The categories for the reporting of AMR are as follows [9]: pAMR 0-negative for pathologic AMR: histopathologic and immunopathologic studies are both negative. pAMR1(H+)-histopathologic AMR alone: histopathologic findings present and immunopathologic findings negative. pAMR1(I+)-immunopathologic AMR alone: histopathologic findings negative and immunopathologic findings positive; that is, CD68+ and/or C4d+ for IHC and C4d+ with or without C3d+ for IF. pAMR2-pathologic AMR: histopathologic and immunopathologic findings are both present. In AMR patients, the inclusion criteria was having at least one positive endomyocardial biopsy (pAMR1 or higher).

The 23 AMR cases were matched to 23 controls by gender, age (±5 years), and follow-up post-transplant. Control patients did not present any distinguishing signs of AMR (pAMR0) or allograft dysfunction. The study protocol was approved by the local Ethics Committee of "*Investigación de Galicia*" (Reference: 2014/012) and the samples were in the “*Colección de muestras para la investigación de insuficiencia cardiaca avanzada y trasplante cardiaco*” of the Instituto de Salud Carlos III (C_0000419, 2012/348). All patients gave written informed consent to participate in the study. The study conforms to the ethical guidelines of the 1975 Declaration of Helsinki.

### Genetic study

DNA was extracted from clots and blood samples using QIAamp DNA Blood Mini Kit (Qiagen Inc. Hilden, Germany), as previously described [8,10]. In all cases and controls, sequencing was performed on NextSeq500 using TruSight One Sequencing Panel (Illumina, San Diego, CA, USA), which included the selected 61 genes related to the biology of B-cells (S1 Table). To assess quality of the NGS data, we define sensitivity as true positive/(true positive+ false negative), specificity as true negative/(true negative+false positive), and accuracy as (true positive+true negative)/(true positive+false positive+ false negative+true negative). To calculate these parameters, direct sequencing of 8 amplicons, containing at least one variant (S2 Table), was performed as previously described [8,10].

### Databases and *in silico* tools used

The potential effect of SNPs associated with the presence or absence of AMR was predicted using *in silico* tools as previously described [8]. Moreover, the minor allele frequency (MAF) of the SNPs detected by NGS was extracted from Single Nucleotide Polymorphism Database (dbSNP) and/or The Exome Aggregation Consortium (ExAC).

### Localization of variants

Topological placement of the mutations was done using the Swissprot database (http://ca.expasy.org/uniprot/). The Uniprot database provides generally accepted residue ranges corresponding with each domain region and specialized subregion.

### Predicting damaging amino acid substitution

Five online tools were used to predict the pathogenicity of the missense variants: SIFT [11] (http://sift.jcvi.org/www/SIFT_seq_submit2.html), Polyphen-2 [12] (http://genetics.bwh.harvard.edu/pph2/), PhDSNP [13] (http://snps.uib.es/phd-snp/phdsnp.html), SNAP2 [14] (https://www.rostlab.org/services/snap/), and MutationTaster [15] (http://www.mutationtaster.org).

### Predicting splice site variants

We evaluated the effect of synonymous variants in the vicinity (±6 pb) of the splice site, including splice site modification and splice-enhancing sequences, using 5 tools: GeneSplicer [16] (http://www.cbcb.umd.edu/software/GeneSplicer/), NetGene2 [17] (http://www.cbs.dtu.dk/services/NetGene2/), ESEfinder 3.0 [18] (http://rulai.cshl.edu/cgi-bin/tools/ESE3/esefinder.cgi), SSP [19] (http://www.fruitfly.org/seq_tools/splice.html), and HSF [20] (http://www.umd.be/HSF/).

### Enzyme-Linked Immunosorbent Assay (ELISA) measurements

Pre-transplant serum samples were used for the determination of functional ITGA4 (n = 16) levels, using a commercially available competitive sandwich ELISA kits (ITGA4 ELISA Kit (Human) (OKEH01078) from Aviva Systems Biology (San Diego, USA)).

ITGA4 measurable concentration range was between 0.156 to 10 ng/mL. Serum samples were measured in duplicate without making any dilution. The absorbance at 450 nm was measured in a spectrophotometer.

### Statistical analyses

Genotype frequencies were tested by multiple inheritance models (codominant, dominant, recessive, log-additive) using SNPStats software [21] and the association function of R software. When these models showed a significant association, McNemar’s chi-square test was performed with R. Moreover, a haplotype association analysis for SNPs described in the same gene was also realized with SNPStats and THESIAS program [22] (http://genecanvas.ecgene.net). In all analysis, P-values≤0.05 were considered to indicate statistical significance.

## Results

### Quality of the NGS data

**Fig 1:**
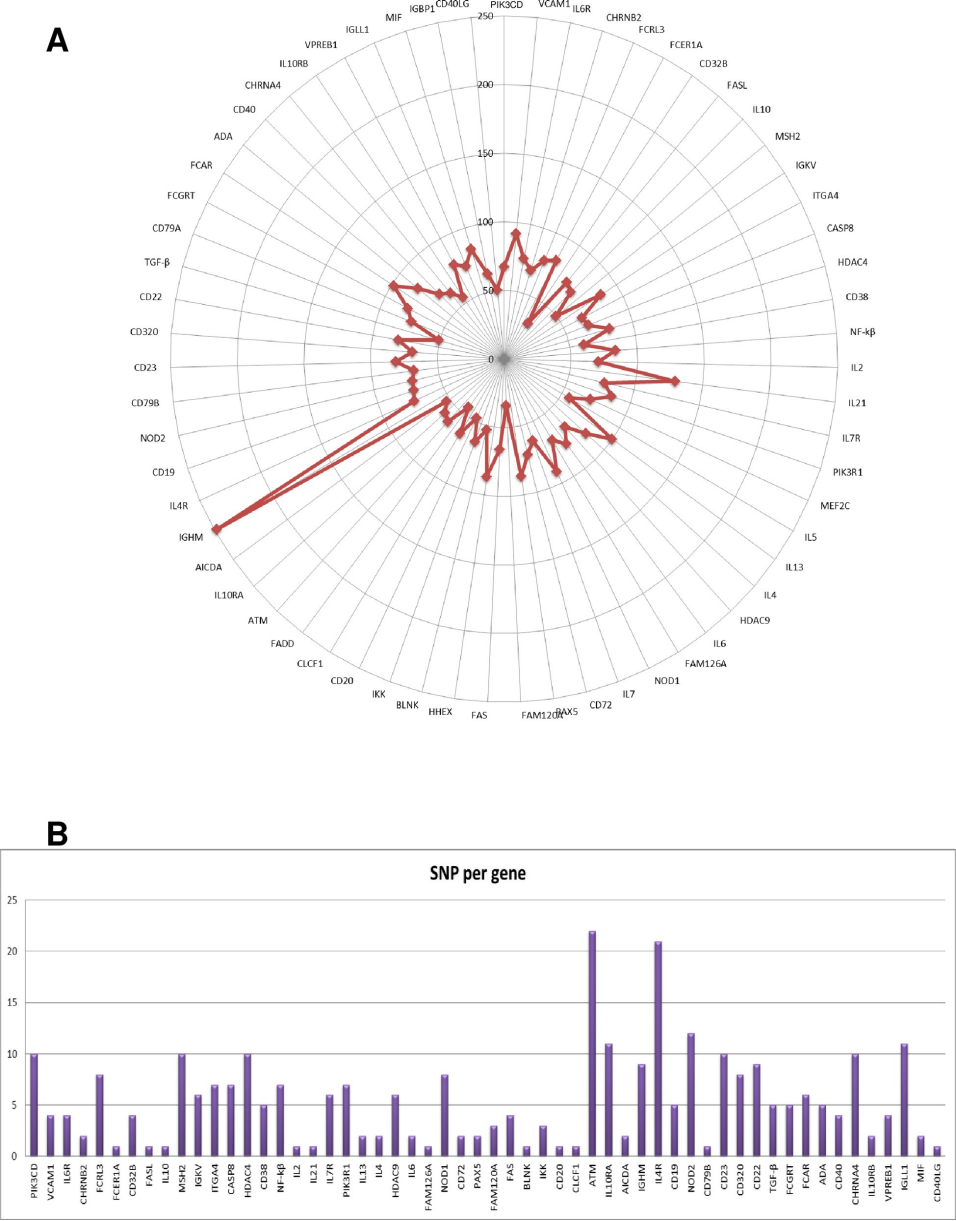
**A, Sequence coverage.** Graph representing the sequence coverage of the genes related to the complement cascade analysed in the study. Over all, mean coverage of the genes studied was 73,5±3,6. **B, SNPs distribution *per* gene.** Bar graph showing the distribution of SNPs *per* gene in AMR (**A**) and control patients (**B**).

The mean coverage over all the genes related to the biology of B-cells studied was 73.5±3.6 fold (Fig 1A). Base calling accuracy, measured by the Phred quality score (Q score), indicates the probability that a given base is called incorrectly by the sequencer. The runs in the NextSeq500 platform showed a Q score > 30, which means a probability of an incorrect base call of 1 in 1000 reads and a base call accuracy 99.9%, in 80.6±2.6% of reads. The range of bases with Q30 score showed in percentage was 75–90% including the five runs performed.

To determine the accuracy, specificity, and sensitivity of the NGS protocol, we directly sequenced 8 selected amplicons containing at least one variant detected by NGS in 13 samples. From 6094 readable bases in the Sanger sequencing, we observed 6067 true negative calls and 27 true positive calls. No false positive or false negative calls were found, together resulting in a sensitivity, specificity, and accuracy of 100%.

### Genotype association to AMR

The AMR patients included in the study, n = 23, and their paired control patients, n = 23, presented the clinical characteristics summarized in Table 1. Fifteen AMR patients diagnosed before 2013 presented the clinical and pathological evidence described in methods section, whereas patients diagnosed after 2013 were classified as: 4 pAMR(I+) and 4 pAMR2.

**Table 1:** Clinical characteristics of the patients included in the study.

Variable	AMR (n = 23 patients)	Control (n = 23 patients)	p-value
Age±sd (years)	50.2±16.5	50.0±15.9	0.8
Male	87.0%(20)	87.0%(20)	1.0
Female	13.0%(3)	13.0%(3)	
Primary heart disease			
Dilated cardiomyopathy	26.1%(6)	43.5%(10)	0.4
Ischemic cardiomyopathy	52.2%(12)	26.1%(6)	
Valvular cardiomyopathy	8.7%(2)	13.0%(3)	
Others	13.0%(3)	17.4%(4)	
Follow-up (years)			
<2 years	17.39%(4)	4.35%(1)	0.2
2–5 years	30.43%(7)	17.39%(4)	
≥6 years	52.17%(12)	78.26%(18)	
Time to AMR diagnosis after transplant (years)	3.5±0.6		
pAMR classification (n = 8)*			
pAMR1(I+)	4		
pAMR2	4		
Immunosupression			
Steroids	100%(23)	100%(23)	0.9
Mycophenolate mofetil	87.0%(20)	95.7%(22)	
Basiliximab	82.6%(19)	95.7%(22)	
Cyclosporine	56.5%(13)	69.6%(16)	
Tacrolimus	65.2%(15)	47.4%(11)	
Everolimus	26.1%(6)	39.1%(9)	
Sandinmune	20.8%(5)	8.3%(2)	
Daclizumab	4.4%(1)	4.4%(1)	
Plasmapheresis	65.2%(15)	0	
Rituximab	65.2%(15)	0	
OKT3	12.5%(3)	0	

* pAMR classification only available for patients who underwent HT after 2013 (n = 8).

After sequencing the whole coding region and the flanking intronic region of 61 genes, we examined the frequency of 285 polymorphisms found in 23 AMR cases and/or in 23 controls (Fig 1B, S3 Table). We found statistically significant differences, after SNPStats and McNemar analysis, in the genotypes of 3 SNPs in the *ITGA4* gene: c.1845G>A, c.2633A>G, and c.2883C>T (Table 2). For c.1845G>ASNP in *ITGA4*, the greatest significance was achieved for a dominant model where A-mutant alleles in homozygotes or heterozygotes (AA/AG) had increased odds of developing AMR compared to the GG homozygotic wild type [p = 0.002; odds ratio (OR) = 7.4; 95% confidence interval1.9–28.9;Table 2]. Moreover, a higher frequency of the A allele in AMR patients was confirmed in the codominant, recessive, and Log-additive model as well as in the McNemar test. For the second *ITGA4* SNP,c.2633A>G, the lowest *P*-value was obtained after applying the dominant model where G-mutant alleles in homozygotes or heterozygotes (GG/AG) had increased odds of developing AMR compared to the AA homozygotic wild type (p<0.001; OR = 9.7; 95% CI 2,4–39.6;Table 2). Additionally, a higher frequency of the G allele in AMR patients was confirmed in the codominant, recessive, overdominant and Log-additive model as well as in the McNemar test. Finally, for the third *ITGA4* SNP,c.2883C>T, the most significant *P*-value was obtained after applying the dominant model where T-mutant alleles in homozygotes or heterozygotes (TT/CT) had increased odds of developing AMR compared to the CC homozygotic wild type (p = 0.002; OR = 7.4; 95% CI 1.9–28.4; Table 2). In addition, a higher frequency of the T allele in AMR patients was confirmed in the codominant and Log-additive model as well as in the McNemar test.

**Table 2:** Statistical analysis of 3 variants in *ITGA4* gene in control and AMR groups.

SNP		Model	Genotype	Control	AMR	OR (95% CI)	p-value	AIC
c.1845G>A	**R (F(x) association)**	Codominant	G/G	14 (60.9%)	4 (17.4%)	1.0	**0.003**	58.0
			G/A	8 (34.8%)	12 (52.2%)	**5.2 (1.3–21.9)**		
			A/A	1 (4.3%)	7 (30.4%)	**24.5 (2.3–262.5)**		
		Dominant	G/G	14 (60.9%)	4 (17.4%)	1.0	**0.002**	58.2
			G/A-A/A	9 (39.1%)	19 (82.6%)	**7.4 (1.9–28.9)**		
		Recessive	G/G-G/A	22 (95.7%)	16 (69.6%)	1.0	**0.01**	61.0
			A/A	1 (4.3%)	7 (30.4%)	**9.6 (1.1–86.2)**		
		Overdominant	G/G-A/A	15 (65.2%)	11 (47.8%)	1.0	0.2	66.0
			G/A	8 (34.8%)	12 (52.2%)	2.0 (0.6–6.7)		
		Log-additive	---	---	---	**5.06 (1.74–14.70)**	**<0.001**	56.0
	**McNemar test**	McNemar Chi squared equals 5.79 with 1 degrees of freedom					**0.02**	
c.2633A>G	**R (F(x) association)**	Codominant	A/A	17 (81.0%)	7 (30.4%)	1.0	**0.001**	53.5
			A/G	4 (19.0%)	13 (56.5%)	**7.9 (1.9–32.8)**		
			G/G	0 (0%)	3 (13.0%)	0.0		
		Dominant	A/A	17 (81.0%)	7 (30.4%)	1.0	**0.001**	53.0
			A/G-G/G	4 (19.0%)	16 (69.6%)	**9.7 (2.4–39.6)**		
		Recessive	A/A-A/G	21 (100%)	20 (87.0%)	1.0	0.2	60.8
			G/G	0 (0%)	3 (13.0%)	0.0		
		Overdominant	A/A-G/G	17 (81.0%)	10 (43.5%)	1.0	**0.009**	58.1
			A/G	4 (19.0%)	13 (56.5%)	**5.5 (1.4–21.7)**		
		Log-additive	---	---	---	**8.6 (2.2–33.3)**	**0.001**	51.7
	**McNemar test**	McNemar Chi squared equals 5.26 with 1 degrees of freedom					**0.02**	
c.2883C>T	**R (F(x) association)**	Codominant	C/C	14 (60.9%)	4 (17.4%)	1.0	**0.006**	59.6
			C/T	7 (30.4%)	12 (52.2%)	**6.0 (1.4–25.6)**		
			T/T	2 (8.7%)	7 (30.4%)	**12.2 (1.8–84.0)**		
		Dominant	C/C	14 (60.9%)	4 (17.4%)	1.0	**0.002**	58.2
			C/T-T/T	9 (39.1%)	19 (82.6%)	**7.4 (1.9–28.9)**		
		Recessive	C/C-C/T	21 (91.3%)	16 (69.6%)	1.0	0.06	64.1
			T/T	2 (8.7%)	7 (30.4%)	4.6 (0.8–25.2)		
		Overdominant	C/C-T/T	16 (69.6%)	11 (47.8%)	1.0	0.1	65.5
			C/T	7 (30.4%)	12 (52.2%)	2.5 (0.7–8.3)		
		Log-additive	---	---	---	**3.9 (1.5–10.4)**	**0.002**	58.2
	**McNemar test**	McNemar Chi squared equals 9.39 with 1 degrees of freedom					**0.002**	

A haplotype association analysis for these 3 SNPs in each subpopulation was then performed (S4 Table). The four observed haplotypes were analyzed: 1.) c.1845G-c.2633A-c.2883C haplotype (GAC); 2.) c.1845A-c.2633G-c.2883T haplotype (AGT), which is a variant with minor allele frequency in the 3 SNPs; 3.) c.1845A-c.2633A-c.2883T haplotype (AAT); and 4.) and c.1845G-c.2633A-c.2883T haplotype (GAT). We found differences in haplotype frequencies to be statistically significant for AGT haplotype (p = 0.005 in SNPstats and p<0.001 in THESIAS) being more frequent in cases (41.3%) than in controls (12.4%). Furthermore, haplotype analysis suggests that the AGT haplotype is associated with the development of AMR with an OR of 11.0 (95% CI 2.3–53.6) and 7.3 (95% CI 2.5–21.3) in SNPstats and THESIAS respectively.

### *In silico* analysis of *ITGA4* variants associated with the presence or absence of AMR in genes related to B-cell biology

Although all the variants associated with the presence or absence of AMR in genes related to B-cell biology found in this study were polymorphisms previously known, we used several *in silico* software in order to predict an impact on the function of the protein (Table 3).

**Table 3:** *ITGA4* variants associated to AMR.

cDNA	dbSNP	Chr 2 position	Protein	1kg_maf	Effect	SIFT	POLYPHEN	PhDSNP	MutationTaster	SNAP2	Grantham score	SSP	HSF	ESEfinder	GENESCAN	NetGene2
c.1845G>A	rs1143674	182374534	p.Thr615(p=)	0.3813 (A)	synonymous	-	-	-	-	-	-	**New splicing site (0**.**9)**	43.2 *vs* 72.2 (+67%)	6.8 vs 7.3 (+16%)	7.2 *vs* 13.1 (+81.5%)	**New splicing site (0**.**8)**
c.2633A>G	rs1143676	182395345	p.Gln878Arg	0.2633 (G)	missense	tolerated(0.61)	benign(0.04)	Neutral	Polymorphism(1)	**Effect(63%)**	43	-	-	-	-	-
c.2883C>T	rs7562325	182399097	p.His961(p=)	0.3737 (T)	synonymous	-	-	-	-	-	-	**0**.**8 *vs* 0**.**6 (-30**.**1%)**	86.6 *vs* 86.8 (+0.2)	7.3 *vs* 8.2 (+12.4%)	**5**.**0 *vs* 4**.**0** **(-20%)**	**0**.**6 *vs* 0**.**3** **(-51**.**6%)**

The missense polymorphism (p.Gln878Arg-*ITGA4*) does not seem to alter the function of the protein (Table 3). In the case of synonymous polymorphisms in the vicinity of splice site (Table 3), we used the Houdayer *et al* criteria of a decrease of at least 20% in the score of the predictions made to consider a possible effect on the splicing process [23]. Only the variant p.His961(p=) in *ITGA4* gene was predicted to have a possible impact on the corresponding natural splice site, by 3 out of 5 software used. However, the synonymous variant p.Thr615(p=) could modify the exonic splicing enhancer, and thus, it could have an impact in protein expression.

### Serum integrin α-4 levels in AMR transplant patients with *ITGA4*-AGT haplotype

**Fig 2:**
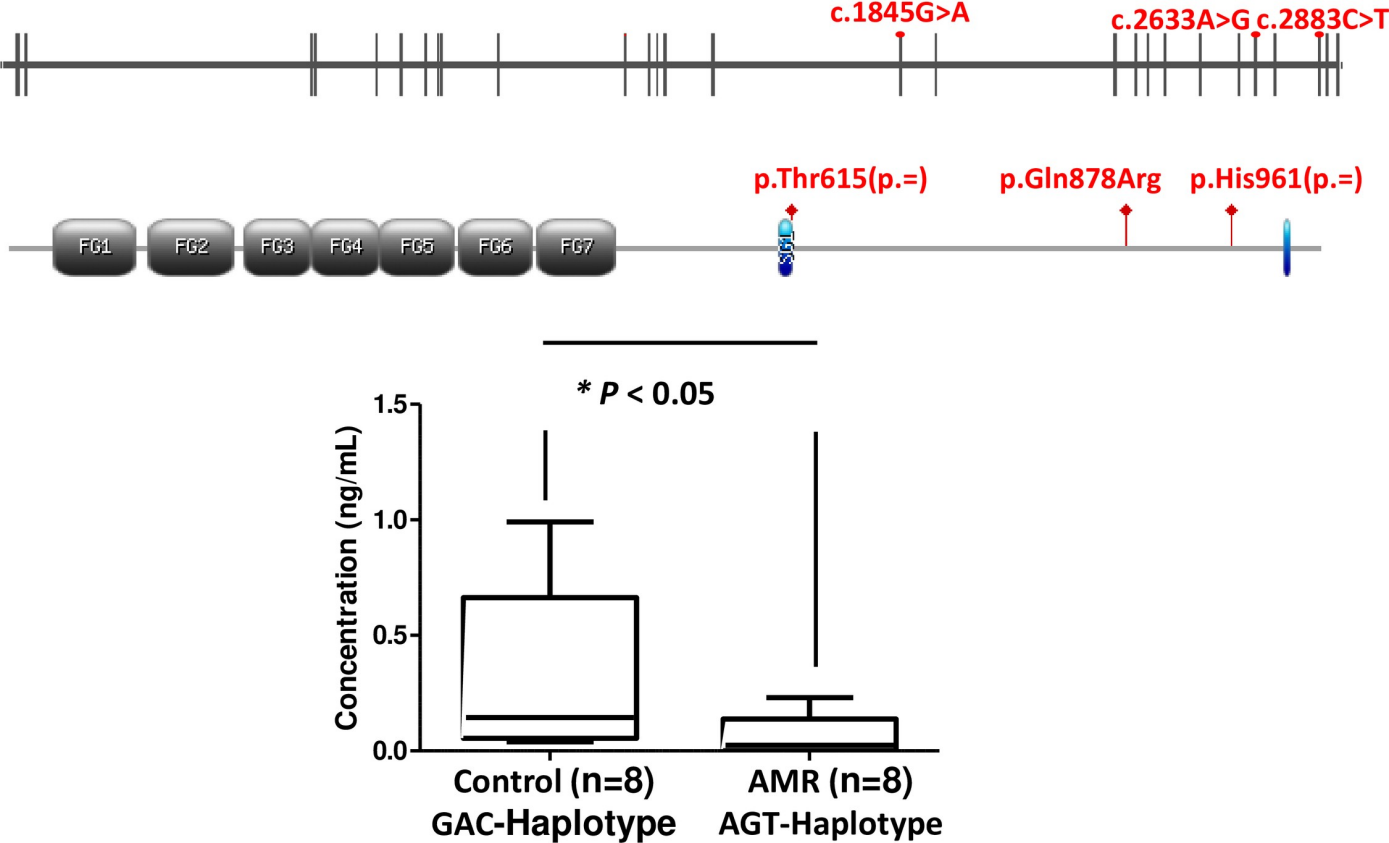
*ITGA4* gene. Schematic structure of the human *ITGA4* gene and primary protein domains of ITGA4 showing relative position of the 3 common polymorphism found (c.1845G>A), c.2633A>G), c.2883C>T). ITGA4 serum levels according to *ITGA4* haplotype among 8 AMR patients (AGT) and 8 control patients (GAC).

The median integrin α-4 serum level measured before transplantation in 8 AMR patients that carried the AGT haplotype was 0.02 ng/mL ([IQR] 0.00–0.1 ng/mL) (Fig 2). This value is significant lower (p<0.05) compared to the median integrin α-4 serum level measured before transplantation in 8 patients who did not present with AMR and in whom the analyzed haplotype was not present (0.1 ng/mL; [IQR] 0.05–0.7 ng/mL).

## Discussion

The present data represents the first study on the effect of variants on genes involved in the B-cell biology related to AMR in HT patients to date. We have identified three SNPs in *ITGA4* gene [p.Thr615(p=), p.Gln878Arg, and p.His961(p=)], which correlates with the development of AMR. These 3 SNPs conform a haplotype: AGT-*ITGA4*, which could be associated with development of AMR in HT patients. Our data showed that the AGT haplotype in *ITGA4* gene in AMR patients present lower levels of integrin α-4 in serum samples compared to the reference GAC haplotype in control patients. In the past decade, efforts to understand how an individual´s genetic can impact on disease susceptibility, severity and responses to treatment have progressed rapidly and the AGT haplotype described in this study could open a new approach to AMR.

Cell adhesion is critical in immune system function, and α4 integrin (also named as CD49d or VLA4), encoded by *ITGA4* gene, plays a particularly prominent role in the immune system through its adhesive and signalling functions [24]. These functions include immune cell trafficking, activation of myeloid cells and naïve T and B lymphocytes, differentiation of effector T cells into Th1, Th2, or Th16, immunological synapse and binding of α4 integrin provides costimulatory signals to T cells [25]. Moreover, α4 integrin mediates B-cell localization in the bone marrow and in germinal centers of secondary lymphoid organs. α4 integrin is also involved in survival signalling pathway that inhibits apoptosis of germinal center B-cells through the up-regulation of the antiapoptotic B-cell lymphoma gene family member, Bcl-XL [24]. The prominent role of integrins in the immune system is also demonstrated because they have been implicated in the pathogenesis of several autoimmune and inflammatory diseases, including asthma, multiple sclerosis, contact hypersensitivity, rheumatoid arthritis, and Crohn´s disease [26–29].

In the field of transplantation, most of the studies focus on integrins were done in CR due to their role in T-cells. It has been described that α_v_β_6_, which belong to another family of integrins but with similar functions to α4 integrin, are increased in acute CR of heart allograft and cardiac allograft vasculopathy in humans [30,31]. In two models of rat kidney transplantation, α_v_ integrin antagonist was able to significantly ameliorate the infiltration of CD8+ T cells and macrophages into the graft and inhibit proliferation of mononuclear cells and fibroblasts in the interstitium of kidney allografts [32]. Treatment with anti-α4 antibody in islet allografts prolongs graft survival, suggesting that the α4 adhesion system may be important in antigen-presentation, T cell activation and lymphocyte recruitment to the graft [33]. Moreover, previous studies have demonstrated that anti-α4 integrin antibody treatment prolongs, but not indefinitely, the survival of cardiac allografts in animal models [34,35].

Our study is the first analysis of *ITGA4* genotype and α4 integrin expression focus on AMR patients. In our cohort, the haplotype AGT present a higher frequency in patients with AMR and it is associated with lower serum levels of α4 integrin. To explain a possible role of α4 integrin in AMR, we have to take into account that α4 integrin are expressed at higher levels by B-cell precursors than mature B-cells [36]. Thus, we can speculate that our lower serum levels in AMR patients could be due the presence of less B-cell precursors and more mature B-cells and these mature B-cells would secrete antibodies that bind to antigens and activate complement, responsible for the AMR [37]. Moreover, Weinländer et al described that inhibition of endothelial cell spreading and migration by inflammatory cytokines is mediated by human guanylate binding protein-1 (GBP-1) through induction of ITGA4 expression [38]. Thus, a down regulation of *ITGA4* gene could have an impact in GBP1 induction, which in turn may result in a higher endothelial cell proliferation and migration during AMR. Furthermore, a down-regulation of the *ITGA4* gene has also been described in other diseases, such as Crohn´s disease [39] or the first stages of B-cell chronic lymphocytic leukemia [40]. Interestingly, in the same line as our haplotype decreases the expression of ITGA4, 2 different SNPs (rs7023923 and rs6740847) downregulate ITGA4 expression (Maugeri et al 2011). However, more research must be done to validate this hypothesis and the role of the AGT haplotype in AMR. If so, these results can open a new approach of the prevention of AMR because natalizumab, a humanized monoclonal antibody against the cell adhesion molecule α4-integrin approved by FDA for multiple sclerosis and Crohn's disease, could be a candidate therapy for preventing AMR.

### Limitations

The current study presents some limitations including a retrospective design, a relatively small sample size, and an evolving criteria of AMR definition. However, we believe that this study opens a new pathway of understanding that variants in genes related to the B cell biology may influence the development of AMR, a field not yet explored.

## Conclusions

The main results of this study are: 1.) AGT haplotype is associated with the development of AMR and 2.) integrin α-4 serum levels in patients carried AGT haplotype are lower that patients carried the reference GAC haplotype. Thus, polymorphisms in the genes related to the biology of B-cells could have an important role in the development of AMR in cardiac transplants. Specifically, the AGT haplotype in *ITGA4* gene could increase the risk of AMR. However, the detailed molecular mechanisms on how this haplotype exerts effects in the immune response leading to AMR are unclear and more research must be done to assess its role.

## Supporting information

S1 Fig The step-wise B cell development and activation. In the bone marrow, development progresses through the pro-B cell and pre-B cell, immature-B cell stages and the most important genes involved are showed. During this differentiation, rearrangements at the immunoglobulin locus result in the generation and surface expression of the pre-B cell receptor and finally a mature B cells that are capable of binding antigen. The 61 genes analysed in this study are showed in bold.
(TIF)

S1 Table Genes involved in the B cell biology analysed in the study. (DOC)

S2 Table Amplicons used to evaluate sensitivity, specificity, and accuracy of NGS technique. (DOC)

S3 Table SNPs found in the genes involved in B cell biology analysed in the study. (DOC)

S4 Table Statistical analysis of the ITGA4 Haplotype association with response. (DOC)

## Abbreviations

AMR: antibody-mediated rejection
CR: cellular rejection
dbSNP: Single Nucleotide Polymorphism Database
ELISA: Enzyme-Linked Immunosorbent Assay
ExAC: The Exome Aggregation Consortium
FN: false-negative
FP: false-positive
HT: heart transplantation
MAF: minor allele frequency
OR: odds ratio
SNPs: single nucleotide polymorphisms
TP: true-positive
TN: true-negative
