# Supplementary figures and images for: AGT haplotype in ITGA4 gene is related to antibody-mediated rejection in heart transplant patients
Source: PLoS One. 2019 Jul 23;14(7):e0219345. doi: 10.1371/journal.pone.0219345 (PMC6650139; doi:10.1371/journal.pone.0219345)

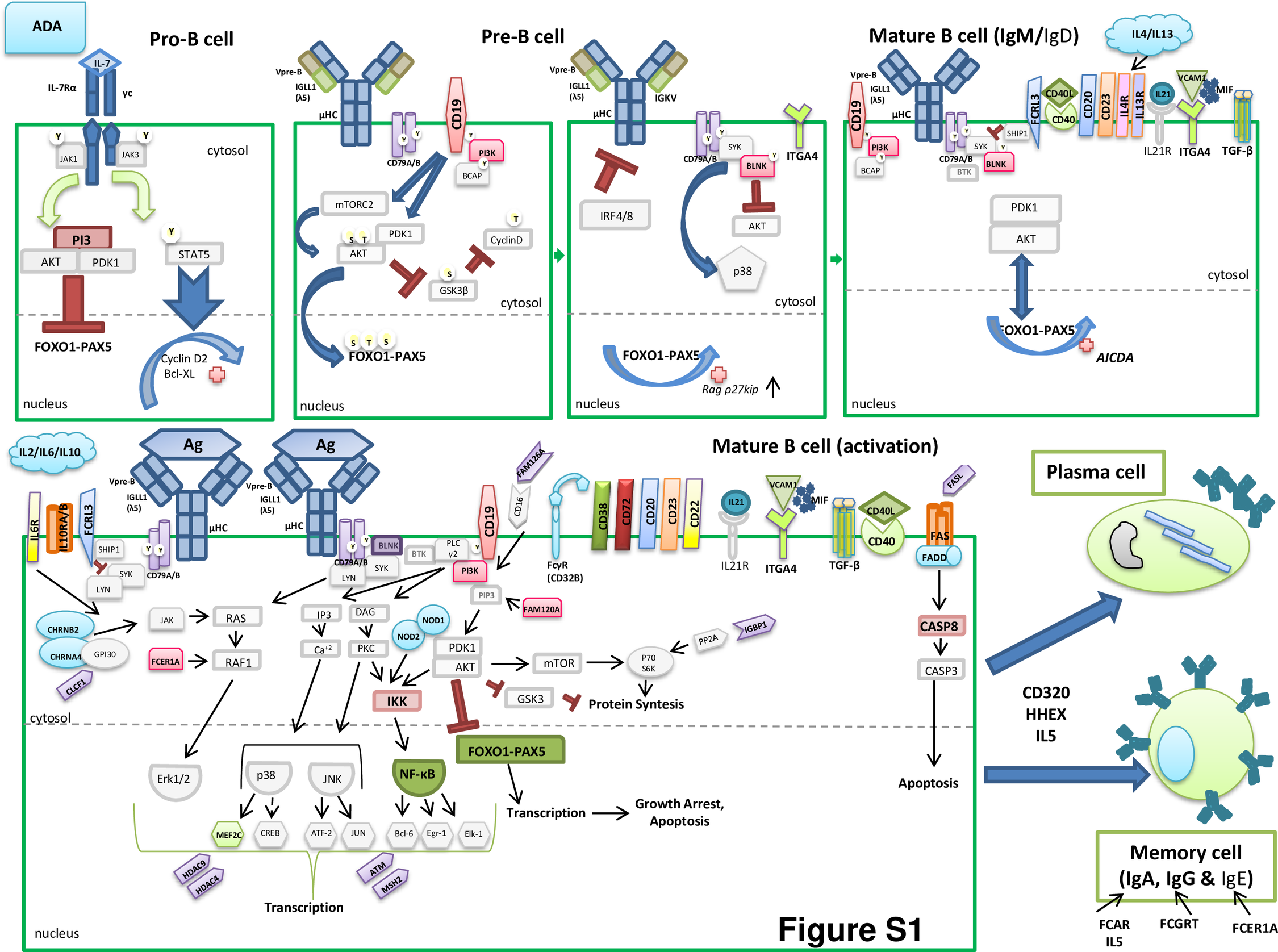

Supplement: S1 Fig — In the bone marrow, development progresses through the pro-B cell and pre-B cell, immature-B cell stages and the most important genes involved are showed. During this differentiation, rearrangements at the immunoglobulin locus result in the generation and surface expression of the pre-B cell receptor and finally a mature B cells that are capable of binding antigen. The 61 genes analysed in this study are showed in bold. (TIF) [file pone.0219345.s001.tif]
