# Supplementary material for: AGT haplotype in ITGA4 gene is related to antibody-mediated rejection in heart transplant patients
Source: PLoS One. 2019 Jul 23;14(7):e0219345. doi: 10.1371/journal.pone.0219345 (PMC6650139; doi:10.1371/journal.pone.0219345)
Supplement: S1 Table — (DOC) [file pone.0219345.s002.doc]

**SUPPLEMENTAL MATERIAL**

**Supplemental Tables**

**S1 Table: Genes involved in the B cell biology analysed in the study.**

| **Gene** | **Position** | **Strand** | **Isoform/Transcript ID** |
| --- | --- | --- | --- |
| ***PIK3CD*** | 1: 9,711,790 - 9,789,173 | POS | NM_005026 /ENST00000377346 |
| ***VCAM1*** | 1:100719640..100739045 | POS | NM_001078/ENST00000294728 |
| ***IL6R*** | 1: 154,377,669 - 154,441,927 | POS | NM_000565/ ENST00000368485 |
| ***CHRNB2*** | 1: 154,540,257 - 154,552,503 | POS | NM_000748/ ENST00000368476 |
| ***FCRL3*** | 1: 157,646,271 - 157,670,648 | NEG | NM_052939/ ENST00000368184 |
| ***FCER1A*** | 1: 159,259,504 - 159,278,015 | POS | NM_002001/ENST00000368115 |
| ***CD32B*** | 1: 161,551,101 - 161,648,445 | POS | NM_004001/ENST00000358671 |
| ***FASL*** | 1: 172,628,154 - 172,636,015 | POS | NM_000639/ ENST00000367721 |
| ***IL10*** | 1: 206,940,947 - 206,945,840 | NEG | NM_000572/ENST00000423557 |
| ***MSH2*** | 2: 47,630,108 - 47,789,451 | POS | NM_000251/ENST00000233146 |
| ***IGKV*** | 2: 89,247,314-89,246,799 | NEG | ENST00000496168 |
| ***ITGA4*** | 2: 182,321,929 - 182,400,915 | POS | NM_000885/ENST00000397033 |
| ***CASP8*** | 2: 202,098,166 - 202,152,435 | POS | NM_001080125/ENST00000358485 |
| ***HDAC4*** | 2: 239,969,864 - 240,323,349 | NEG | NM_006037/ENST00000345617 |
| ***CD38*** | 4: 15,779,898 - 15,851,070 | POS | NM_001775/ENST00000226279 |
| ***NF-k*β** | 4: 102,501,329-102,617,302 | POS | NM_003998/ENST00000226574 |
| ***IL2*** | 4: 123,372,625 - 123,377,881 | NEG | NM_000586/ ENST00000226730 |
| ***IL21*** | 4: 123,533,783 - 123,542,225 | NEG | NM_021803/ENST00000264497 |
| ***IL7R*** | 5: 35856875-35879603 | POS | NM_002185/ENST00000303115 |
| ***PIK3R1*** | 5: 67,511,548 - 67,597,650 | POS | NM_181523 /ENST00000521381 |
| ***MEF2C*** | 5: 88,013,975 - 88,199,923 | NEG | NM_002397 /ENST00000504921 |
| ***IL5*** | 5: 131,877,136 - 131,892,531 | NEG | NM_000879/ENST00000231454 |
| ***IL13*** | 5: 131,991,955 - 131,996,803 | POS | NM_002188/  ENST00000304506 |
| ***IL4*** | 5: 132,009,678 - 132,018,369 | POS | NM_000589/ENST00000231449 |
| ***HDAC9*** | 7: 18,126,572 - 19,036,994 | POS | NM_178425/ENST00000441542 |
| ***IL6*** | 7: 22,765,503 - 22,771,622 | POS | NM_000600/ ENST00000258743 |
| ***FAM126A*** | 7: 22,980,878 - 23,053,750 | NEG | NM_032581/ENST00000432176 |
| ***IL7*** | 8: 78,675,870 - 78,805,523 | NEG | NM_000880/ENST0000026385 |
| ***CD72*** | 9: 35,609,530 - 35,646,808 | NEG | NM_001782/ENST00000259633 |
| ***PAX5*** | 9: 36,833,272 - 37,034,104 | NEG | NM_016734 /ENST00000358127 |
| ***FAM120A*** | 9: 96,214,004 - 96,328,398 | POS | NM_014612/ENST00000277165 |
| ***FAS*** | 10: 90,750,414 - 90,775,543 | POS | NM_000043/ ENST00000355740 |
| ***HHEX*** | 10: 94,447,945 - 94,455,404 | POS | NM_002729/ENST00000282728 |
| ***BLNK*** | 10: 97,951,458 - 98,031,345 | NEG | NM_013314/ENST00000224337 |

| **Gene** | **Position** | **Strand** | **Isoform/Transcript ID** |
| --- | --- | --- | --- |
| ***IKK*** | 10: 101,948,055 - 101,989,377 | NEG | NM_001278/ ENST00000370397 |
| ***CD20*** | 11: 60,223,225 - 60,238,234 | POS | NM_152866 /ENST00000534668 |
| ***CLCF1*** | 11: 67,131,639 - 67,141,649 | NEG | NM_013246/ENST00000312438 |
| ***FADD*** | 11: 70,203,163 - 70,207,402 | POS | NM_003824/ENST00000301838 |
| ***ATM*** | 11: 108,093,211 - 108,239,830 | POS | NM_000051/ ENST00000278616 |
| ***IL10RA*** | 11: 117,857,063 - 117,872,197 | POS | NM_001558/ ENST00000227752 |
| ***AICDA*** | 12: 8,754,762 - 8,765,468 | NEG | NM_020661/ ENST00000229335 |
| ***IGHM*** | 14: 106,320,349 - 106,322,324 | NEG | ENST00000390559 |
| ***IL4R*** | 16: 27,324,989 - 27,376,100 | POS | NM_000418/  ENST00000395762 |
| ***CD19*** | 16: 28,943,260 - 28,950,668 | POS | NM_001178098/ENST00000538922 |
| ***CD79B*** | 17: 62,006,100 - 62,009,715 | NEG | NM_001039933/ENST00000392795 |
| ***CD23*** | 19: 7,753,644 - 7,767,033 | NEG | NM_002002/ENST00000346664 |
| ***CD320*** | 19: 8,367,011 - 8,373,241 | NEG | NM_016579/ENST00000301458 |
| ***CD22*** | 19: 35,810,164 - 35,838,259 | POS | NM_001771/ ENST00000085219 |
| ***TGF-*β** | 19: 41,807,492 - 41,859,817 | NEG | NM_000660 /ENST00000221930 |
| ***CD79A*** | 19: 42,381,190 - 42,385,440 | POS | NM_001783/ ENST00000221972 |
| ***FCGRT*** | 19: 50,010,073 - 50,029,591 | POS | NM_001136019/ENST00000221466 |
| ***FCAR*** | 19: 55,385,704 - 55,401,839 | POS | NM_002000 / ENST00000355524 |
| ***ADA*** | 20: 43,248,163 - 43,280,875 | NEG | NM_000022 / ENST00000372874 |
| ***CD40*** | 20: 44,746,911 - 44,758,503 | POS | NM_001250 /ENST00000372285 |
| ***CHRNA4*** | 20: 61,975,420 - 62,009,754 | NEG | NM_000744/ ENST00000370263 |
| ***IL10RB*** | 21: 34,638,663 - 34,669,540 | POS | NM_000628/ ENST00000290200 |
| ***VPREB1*** | 22: 22,599,087 - 22,599,928 | POS | NM_007128/ENST00000403807 |
| ***IGLL1*** | 22: 23,915,312 - 23,922,496 | NEG | NM_020070 /ENST00000330377 |
| ***MIF*** | 22: 24,236,191 - 24,237,415 | POS | NM_002415/ENST00000215754 |
| ***IGBP1*** | X: 69,353,299 - 69,386,175 | POS | NM_001551 /ENST00000356413 |
| ***CD40LG*** | X: 135,730,352 - 135,742,550 | POS | NM_000074 / ENST00000370629 |
