# Supplementary material for: AGT haplotype in ITGA4 gene is related to antibody-mediated rejection in heart transplant patients
Source: PLoS One. 2019 Jul 23;14(7):e0219345. doi: 10.1371/journal.pone.0219345 (PMC6650139; doi:10.1371/journal.pone.0219345)
Supplement: S2 Table — (DOC) [file pone.0219345.s003.doc]

**S2 Table: Amplicons used to evaluate sensitivity, specificity, and accuracy of NGS technique**.

| **Gene** | **Reference sequence** | **FRAGMENT** | **Primer sequence (5’🡪3’)** | **Amplicon size (nt)** | **Samples *per* amplicon** |
| --- | --- | --- | --- | --- | --- |
| *CD38* | NC_000004.12 (15778265..15853243) | CD38Ex6_F | aggtcctagccagtgccttt | 325 | 1 |
|  |  | CD38Ex6_R | tgttgggattcatgcacatt |  |  |
| *MEF2C* | NC_000005.10 (88718241..88904105) | MEF2CEx5_F | tggaaaaataagggctgtctg | 398 | 1 |
|  |  | MEF2CEx5_R | ggatggtaagccatgaagga |  |  |
| *ITGA4* | NC_000002.12 (181456892..181538928) | ITGA4Ex11_F | tttcgaagttgcattctttgt | 234 | 1 |
|  |  | ITGA4Ex11_R | aagtccacataaaccctggaa |  |  |
| *ATM* | NC_000011.10 (108222484..108369102) | ATMEx43_F | caccacacccagctgatattt | 586 | 1 |
|  |  | ATMEx43_R | gcctaggtgcattaactgctt |  |  |
| *IGLL1* | NC_000022.11 (23573125..23580548) | IGLL1Ex1_F | gaccagggcaccactctcta | 388 | 1 |
|  |  | IGLL1Ex1_R | cctccagggattaaccttcc |  |  |
| *FCER2* | NC_000019.10 (7688757..7702755) | FCER2Ex11_F | gagaagggagggtgcagac | 400 | 2 |
|  |  | FCER2Ex11_R | tcagccacaaagaggctttta |  |  |
|  |  | FCER2Ex9+10_F | gcccaaggcacttccatt | 499 | 4 |
|  |  | FCER2Ex9+10_R | tccacggtatttccatctcc |  |  |
| *IL4R* | NC_000016.10 (27313668..27364778) | IL4REx4_F | ctcaggctgctcctgtgtct | 240 | 1 |
|  |  | IL4REx4_R | ctggacccagctcactcttg |  |  |
| *IL6R* | NC_000001.11 (154405193..154469450) | IL6REx2_F | tgtcttctccctcctccaga | 373 | 1 |
|  |  | IL6REx2_R | caaggaaatggtgggagttt |  |  |

The reference sequences were extracted from GenBank-GRCh37.p9
