# Supplementary material for: AGT haplotype in ITGA4 gene is related to antibody-mediated rejection in heart transplant patients
Source: PLoS One. 2019 Jul 23;14(7):e0219345. doi: 10.1371/journal.pone.0219345 (PMC6650139; doi:10.1371/journal.pone.0219345)
Supplement: S3 Table — (DOC) [file pone.0219345.s004.doc]

**S3 Table: SNPs found in the genes involved in B cell biology analysed in the study.**

| **gene** | **dbsnp** | **ref** | **genotype** | **allele** | **1kg_maf** | **Nº patients** | **effect** | **HGVS-prot** |
| --- | --- | --- | --- | --- | --- | --- | --- | --- |
| *PIK3CD* | rs150205370 | G | G/A | A | 0.000008 (A) | 1HZ | missense_variant | p.Val98Ile |
| *PIK3CD* | rs113176101 | C | C/T | T | 0.0234 (T) | 1HZ | intron_variant | IVS4-3C>T |
| *PIK3CD* | rs2230735 | A | A/G | G | 0.0453 (G) | 2HZ | synonymous_variant | p.Thr226(p=) |
| *PIK3CD* | rs145697393 | G | G/A | A | 0.0041 (A) | 1HZ | synonymous_variant | p.Pro236(p=) |
| *PIK3CD* | rs557471275 | T | T/C | C | 0.0001 C | 1HZ | missense_variant | p.Met285Thr |
| *PIK3CD* | rs28730668 | G | G/A | A | 0.0220 (A) | 1HZ | missense_variant | p.Val296Ile |
| *PIK3CD* | rs28730669 | G | G/C | C | 0.0096 (C) | 3HZ | intron_variant | IVS7-9G>C |
| *PIK3CD* | rs61755420 | C | C/G | G | 0.0128 (G) | 5HZ | missense_variant | p.Ser312Cys |
| *PIK3CD* | rs139848768 | C | C/T | T | 0.0037 (T) | 2HZ | synonymous_variant | p.Ser773(p=) |
| *PIK3CD* | rs11121484 | C | C/T | T | 0.2294 (T) | 2HM/4HZ | synonymous_variant | p.Tyr936(p=) |
| *VCAM1* | rs2392221 | C | C/T | T | 0.1474 (T) | 8HZ | intron_variant | IVS3-7C>T |
| *VCAM1* | rs114207303 | C | C/G | G | 0.0037 (G) | 1HZ | missense_variant | p.Leu555Val |
| *VCAM1* | rs3176878 | C | C/T | T | 0.1218 (T) | 1HM/10HZ | synonymous_variant | P.Asp693(p=) |
| *VCAM1* | rs3176879 | A | A/G | G | 0.0626 (G) | 1HZ | synonymous_variant | p.Lys736(p=) |
| *IL6R* | rs2228144 | G | G/A | A | 0.1190 (A) | 14HZ | synonymous_variant | p.Ala37(p=) |
| *IL6R* | rs2229237 | C | C/T | T | 0.0220 (T) | 5HZ | synonymous_variant | p.His70(p=) |
| *IL6R* | rs2228145 | A | A/C | C | 0.3196 (C) | 8HM/15HZ | missense_variant | p.Asp358Ala |
| *IL6R* | rs2228146 | G | G/A | A | 0.0531 (A) | 2HZ | missense_variant | p.Val385Ile |
| *CHRNB2* | rs3926124 | A | A/G | G | 0.0957 (G) | 2HZ | intron_variant | IVS2+9A>G |
| *CHRNB2* | rs55857552 | G | G/A | A | 0.0013 (A) | 2HZ | synonymous_variant | p.Ala411(p=) |
| *FCRL3* | rs2282284 | T | T/C | C | 0.0536 (C) | 3HZ | missense_variant | p.Asn721Ser |
| *FCRL3* | rs944627 | G | G/A | A | 0.0339 (A) | 2HZ | missense_variant | p.Pro660Leu |
| *FCRL3* | rs4319323 | T | T/C | C | 0.0325 (C) | 2HZ | synonymous_variant | p.Pro643(p=) |
| *FCRL3* | rs71630672 | G | G/A | A | 0.0009 (A) | 1HZ | stop_gained | p.Gln121Ter |
| *FCRL3* | rs7522061 | T | T/C | C | 0.4876 (T) | 11HM/12HZ | missense_variant | p.Asn28Asp |
| *FCRL3* | rs12140725 | G | A/A | A | 0.1735 (A) | 5HM/4HZ | intron_variant | IVS3-8C>T |
| *FCRL3* | rs113752575 | CGGCC | CGGCC/TGGCG | TGGCG | 0.0215 (T) | 2HZ | 5_prime_UTR_variant | 5'UTR-7delGGCCGinsCGCCA |
| *FCRL3* | rs144282361 | C | C/T | T | 0.0018 (T) | 3HZ | 5_prime_UTR_variant | 5'UTR-10G>A |
| *FCER1A* | rs41264475 | C | C/A | A | 0.0096 (A) | 2HZ | missense_variant | p.Asn247Lys |
| *CD32B* | rs5017568 | G | G/A | A | 0.0054 (A) | 2HZ | synonymous_variant | p.Gln63(p=) |
| *CD32B* | rs144573139 | G | G/A | A | 0.0055 (A) | 1HZ | synonymous_variant | p.Gly73(p=) |
| *CD32B* | rs6665610 | G | G/A | A | 0.1504 (A) | 9HZ | synonymous_variant | p.Thr112(p=) |
| *CD32B* | rs182968886 | G | G/A | A | 0.0975 (A) | 1HM/8HZ | synonymous_variant | p.Leu204(p=) |
| *FASLG* | rs61756244 | G | G/A | A | 0.0458 (A) | 1HZ | synonymous_variant | p.Pro49(p=) |
| *IL10* | rs200783164 | G | G/T | T | 0.0009 (T) | 1HZ | missense_variant | p.Leu71Met |
| *MSH2* | rs2303426 | C | G/G | G | 0.3846 (C) | 12HM/10HZ | intron_variant | IVS1+9C>G |
| *MSH2* | rs35898375 | G | G/A | A | 0.0018 (A) | 1HZ | synonymous_variant | p.Lys113(p=) |
| *MSH2* | rs17217772 | A | A/G | G | 0.0188 (G) | 1HZ | missense_variant | p.Asn127Ser |
| *MSH2* | rs587779193 | G | G/A | A |  | 2HZ | intron_variant | IVS5+1G>A |
| *MSH2* | rs4987188 | G | G/A | A | 0.0092 (A) | 2HZ | missense_variant | p.Gly322Asp |
| *MSH2* | rs63750505 | G | G/A | A | 0.0009 (A) | 1HZ | synonymous_variant | p.Gln324(p=) |
| *MSH2* | rs17224360 | T | T/C | C | 0.0073 (C) | 1HM/3HZ | intron_variant | IVS6-10T>C |
| *MSH2* | rs12998837 | A | A/T | T | 0.0650 (T) | 9HZ | intron_variant | IVS9-9A>T |
| *MSH2* | rs61756466 | T | T/C | C | 0.0032 (C) | 2HZ | synonymous_variant | p.Leu556(p=) |
| *MSH2* | rs2303428 | T | T/C | C | 0.1282 (C) | 1HM/9HZ | intron_variant | IVS12-6T>C |
| *IGKV* | rs201861379 | T | T/C | C | 0.0747( C) | 5HZ | synonymous_variant | p.Leu76(p=) |
| *IGKV* | rs386648262 | AGACGCCTT | AGACGCCTT/GGAGGCATC | GGAGGCATC | 0.07473(G) | 5HZ | missense_variant | p.Lys72Asp |
| *IGKV* | rs10865492 | C | C/T | T | 0.1519 (T) | 10HZ | synonymous_variant | p.Ala73(p=) |
| *IGKV* | rs200979873 | T | T/G | G | 0.0050 (G) | 1HZ | missense_variant | p.Lys64Asn |
| *IGKV* | rs201626492 | A | A/G | G | 0.0014 (G) | 1HZ | synonymous_variant | p.Leu55(p=) |
| *IGKV* | rs112599788 | G | G/A | A | 0.0362 (A) | 5HZ | 5_prime_UTR_variant | 5'UTR-16C>T |
| *ITGA4* | rs35419274 | G | G/A | A | 0.0005 (A) | 3HZ | missense_variant | p.Val359Ile |
| *ITGA4* | rs1143674 | G | G/A | A | 0.3813 (A) | 8HM/20HZ | synonymous_variant | p.Thr615(p=) |
| *ITGA4* | rs35322532 | T | T/A | A | 0.0110 (A) | 1HZ | missense_variant | p.Ser634Thr |
| *ITGA4* | rs1143675 | T | T/C | C | 0.0023 (C) | 1HZ | missense_variant | p.Val824Ala |
| *ITGA4* | rs1143676 | A | A/G | G | 0.2633 (G) | 3HM/17HZ | missense_variant | p.Gln878Arg |
| *ITGA4* | rs7562325 | C | C/T | T | 0.3737 (T) | 9HM/18HZ | synonymous_variant | p.His961(p=) |
| *ITGA4* | rs61731209 | C | C/T | T | 0.0027 (T) | 1HZ | synonymous_variant | p.Asn1026(p=) |
| *CASP8* | rs3769824 | T | T/C | C | 0.0298 (C) | 4HZ | initiator_codon_variant | p.Met1Thr |
| *CASP8* | rs3769823 | A | A/G | G | 0.3484 (A) | 24HM/21HZ | missense_variant | p.Lys14Arg |
| *CASP8* | rs534884641 | CG | CG/C | - | 0.0002 (T) | 1HZ | frameshift_variant | p.Asp129IlefsTer4 |
| *CASP8* | rs17860422 | C | C/T | T | 0.0023 (T) | 1HZ | synonymous_variant | p.Ser172(p=) |
| *CASP8* | rs1045485 | G | G/C | C | 0.0678 (C) | 1HM/9HZ | missense_variant | p.Asp344His |
| *CASP8* | rs779189628 | A | A/G | G | 0.000008 (G) | 1HZ | missense_variant | p.Gln350Arg |
| *CASP8* | rs1045487 | G | G/A | A | 0.1488 (A) | 6HZ | synonymous_variant | p.Gln379(p=) |
| *HDAC4* | rs61754649 | G | G/A | A | 0.0142 (A) | 2HZ | synonymous_variant | p.Ser874(p=) |
| *HDAC4* | rs1063639 | G | G/A | A | 0.4199 (G) | 14HM/25HZ | synonymous_variant | p.Pro855(p=) |
| *HDAC4* | rs61752234 | T | T/C | C | 0.0366 (C) | 1HZ | synonymous_variant | p.Thr787(p=) |
| *HDAC4* | rs7585225 | T | C/C | C | 0.1777 (T) | 34HM/12HZ | intron_variant | IVS15+10A>G |
| *HDAC4* | rs79199572 | C | C/T | T | 0.0234 (T) | 2HZ | synonymous_variant | p.Pro522(p=) |
| *HDAC4* | rs10207474 | T | T/C | C | 0.0847 (C) | 1HZ | intron_variant | IVS11+10A>G |
| *HDAC4* | rs35291459 | C | C/T | T | 0.0788 (T) | 4HZ | synonymous_variant | p.Pro423(p=) |
| *HDAC4* | rs2290087 | A | G/G | G | 0.1378 (A) | 33HM/11HZ | intron_variant | IVS7-7T>C |
| *HDAC4* | rs148880349 | G | G/A | A | 0.0006 (A) | 1HZ | synonymous_variant | p.His227(p=) |
| *HDAC4* | rs2290092 | C | T/T | T | 0.1021 (T) | 2HM/7HZ | intron_variant | IVS4+8G>A |
| *CD38* | rs78580781 | C | C/T | T | 0.0027 (T) | 1HZ | synonymous_variant | p.Tyr70(p=) |
| *CD38* | rs1800561 | C | C/T | T | 0.0018 (T) | 1HZ | missense_variant | p.Arg140Trp |
| *CD38* | rs1800051 | A | A/C | C | 0.0888 (C) | 5HZ | synonymous_variant | p.Ile168(p=) |
| *CD38* | rs950566 | A | A/G | G | 0.0110 (G) | 1HZ | intron_variant | IVS5+5A>G |
| *CD38* | rs139655522 | A | A/G | G | 0.0000 (G) | 1HZ | synonymous_variant | p.Arg247(p=) |
| *NF-kβ* | rs4648039 | C | C/T | T | 0.0087 (T) | 4HZ | synonymous_variant | p.Tyr350(p=) |
| *NF-kβ* | rs1609993 | T | C/C | C | 0.0417 (T) | 38HM/7HZ | synonymous_variant | p.Aala381(p=) |
| *NF-kβ* | rs4648072 | A | A/G | G | 0.0119 (G) | 1HZ | missense_variant | p.Met507Val |
| *NF-kβ* | rs190121351 | T | T/C | C | 0.0005 (C) | 1HZ | synonymous_variant | p.Asp581(p=) |
| *NF-kβ* | rs4648093 | G | G/A | A | 0.0243 (A) | 1HZ | synonymous_variant | p.Thr585(p=) |
| *NF-kβ* | rs777416263 | C | C/T | T | 0.00002 (T) | 1HZ | intron_variant | IVS20+9C>T |
| *NF-kβ* | rs148268461 | T | T/TA | A | 0.1021 (A) | 4HZ | intron_variant | IVS23+2_2749+3insA |
| *IL2* | rs2069763 | C | A/A | A | 0.3443 (A) | 4HM/18HZ | synonymous_variant | p.Leu38(p=) |
| *IL21* | rs4833837 | G | A/A | A | 0.1992 (G) | 25HM/16HZ | synonymous_variant | p.Cys78(p=) |
| *IL7R* | rs1494558 | T | T/C | C | 0.3869 (T) | 17HM/24HZ | missense_variant | p.Ile66Thr |
| *IL7R* | rs1494555 | G | G/A | A | 0.3260 (G) | 18HM/23HZ | missense_variant | p.Val138Ile |
| *IL7R* | rs2228141 | C | C/T | T | 0.1346 (T) | 1HM/8HZ | synonymous_variant | p.His165(p=) |
| *IL7R* | rs6897932 | C | C/T | T | 0.1909 (T) | 3HM/12HZ | missense_variant | p.Thr244Ile |
| *IL7R* | rs41270321 | A | A/C | C | 0.0078 (C) | 1HZ | missense_variant | p.Asn348Thr |
| *IL7R* | rs3194051 | A | A/G | G | 0.2257 (G) | 4HM/16HZ | missense_variant | p.Ile356Val |
| *PIK3R1* | rs706713 | C | C/T | T | 0.4414 (T) | 3HM/20HZ | synonymous_variant | p.Tyr73(p=) |
| *PIK3R1* | rs376498902 | T | T/C | C | 0.00002 ( C) | 1HZ | intron_variant | IVS2+9T>C |
| *PIK3R1* | rs375394677 | C | C/A | A | 0.0004 (A) | 1HZ | intron_variant | IVS3+5C>A |
| *PIK3R1* | rs61749601 | T | T/C | C | 0.0156 (C) | 2HZ | synonymous_variant | p.Ile207(p=) |
| *PIK3R1* | rs3730089 | G | G/A | A | 0.2175 (A) | 8HZ | missense_variant | p.Met326Ile |
| *PIK3R1* | rs3730090 | C | C/T | T | 0.0695 (T) | 1HZ | synonymous_variant | p.Phe392(p=) |
| *PIK3R1* | rs3729981 | T | T/C | C | 0.0435 (C) | 1HM/1HZ | synonymous_variant | p.Leu703(p=) |
| *IL13* | rs55733734 | C | C/T | T | 0.0009 (T) | 2HZ | synonymous_variant | p.Thr20(p=) |
| *IL13* | rs20541 | A | G/G | G | 0.2683 (A) | 32HM/13HZ | missense_variant | p.Gln144Arg |
| *IL4* | rs35648164 | C | C/T | T | 0.0050 (T) | 1HZ | synonymous_variant | p.Asp111(p=) |
| *IL4* | rs2243290 | C | C/A | A | 0.4066 (A) | 15HZ | intron_variant | IVS3-9C>A |
| *HDAC9* | rs34096894 | C | C/T | T | 0.0408 (T) | 2HZ | synonymous_variant | p.Leu194(p=) |
| *HDAC9* | rs150067220 | G | G/A | A | 0.0069 (A) | 1HZ | intron_variant | IVS7+10G>A |
| *HDAC9* | rs35691757 | C | C/T | T | 0.0311 (T) | 1HZ | synonymous_variant | p.Ser527(p=) |
| *HDAC9* | rs1178127 | A | A/G | G | 0.3301 (G) | 2HM/23HZ | synonymous_variant | p.Pro624(p=) |
| *HDAC9* | rs17139769 | A | A/C | C | 0.0586 (C) | 1HZ | intron_variant | IVS12+6A>C |
| *HDAC9* | rs2389998 | C | C/T | T | 0.2312 (C) | 29HM/16HZ | synonymous_variant | p.Phe1010(p=) |
| *IL6* | rs2069860 | A | A/T | T | 0.0018 (T) | 1HZ | missense_variant | p.Asp162Val |
| *IL6* | rs2069849 | C | C/T | T | 0.0559 (T) | 2HZ | synonymous_variant | p.Phe201(p=) |
| *FAM126A* | rs3735231 | T | T/C | C | 0.3768 (C) | 5HM/11HZ | synonymous_variant | p.Ser208(p=) |
| *CD72* | rs34791102 | G | G/A | A | 0.0517 (A) | 3HZ | missense_variant | p.Pro234Leu |
| *CD72* | rs35185125 | C | C/A | A | 0.0124 (A) | 2HZ | missense_variant | p.Val50Leu |
| *PAX5* | rs3780135 | G | A/A | A | 0.2138 (G) | 39HM/5HZ | synonymous_variant | p.Tyr370(p=) |
| *PAX5* | rs35469494 | C | C/T | T | 0.0174 (T) | 5HZ | synonymous_variant | p.Gly343(p=) |
| *FAM120A* | rs10821135 | C | T/T | T | 0.4144 (C) | 11HM/19HZ | synonymous_variant | p.His254(p=) |
| *FAM120A* | rs35062269 | T | T/TA | A | 3.594e-05 (A) | 7HZ | intron_variant | IVS5+2_933+3insA |
| *FAM120A* | rs147979681 | C | C/T | T | 0.0713 (T) | 1HM/4HZ | synonymous_variant | p.His447(p=) |
| *FAS* | rs5030766 | A | A/G | G | 0.0266 (G) | 1HZ | 5_prime_UTR_variant | 5'UTR-34A>G |
| *FAS* | rs3218619 | G | G/A | A | 0.0343 (A) | 1HZ | missense_variant | p.Ala16Thr |
| *FAS* | rs2229521 | A | A/G | G | 0.0394 (G) | 3HZ | synonymous_variant | p.Thr74(p=) |
| *FAS* | rs2234978 | T | T/C | C | 0.2358 (T) | 22HM/19HZ | synonymous_variant | p.Thr214(p=) |
| *BLNK* | rs727852 | A | A/G | G | 0.4876 (A) | 28HM/17HZ | synonymous_variant | p.Pro57(p=) |
| *IKK* | rs17880383 | G | G/A | A | 0.0394 (A) | 6HZ | synonymous_variant | p.Ser486(p=) |
| *IKK* | rs34458357 | T | T/C | C | 0.0014 (C) | 1HZ | synonymous_variant | p.Gly447(p=) |
| *IKK* | rs2230804 | C | T/T | T | 0.4377 (C) | 21HM/10HZ | missense_variant | p.Val268Ile |
| *CD20* | rs2070770 | C | C/T | T | 0.0678 (T) | 1HM/4HZ | synonymous_variant | p.Ile72(p=) |
| *CLCF1* | rs867193 | A | T/T | T | 0.0852 (T) | 1HM/2HZ | synonymous_variant | p.Pro36(p=) |
| *ATM* | rs1800054 | C | C/G | G | 0.0055 (G) | 1HZ | missense_variant | p.Ser49Cys |
| *ATM* | rs2234997 | T | T/A | A | 0.0458 (A) | 1HZ | missense_variant | p.Asp126Glu |
| *ATM* | rs3218707 | G | G/C | C | 0.0124 (C) | 1HZ | missense_variant | p.Val182Leu |
| *ATM* | rs3218674 | C | C/T | T | 0.0069 (T) | 1HZ | synonymous_variant | p.Val245(p=) |
| *ATM* | rs28904919 | C | C/T | T | 0.0014 (T) | 1HZ | missense_variant | p.Ser333Phe |
| *ATM* | rs138398778 | C | C/T | T | 0.00009 (T) | 1HZ | missense_variant | p.Arg337Cys |
| *ATM* | rs4986761 | T | T/C | C | 0.0050 (C) | 1HZ | missense_variant | p.Ser707Pro |
| *ATM* | rs34231402 | T | T/A | A | 0.0005 (A) | 1HZ | missense_variant | p.Phe763Leu |
| *ATM* | rs3218695 | C | C/A | A | 0.0092 (A) | 1HZ | missense_variant | p.Asp814Glu |
| *ATM* | rs1800056 | T | T/C | C | 0.0069 (C) | 1HZ | missense_variant | p.Phe858Leu |
| *ATM* | rs1800057 | C | C/G | G | 0.0151 (G) | 4HZ | missense_variant | p.Pro1054Arg |
| *ATM* | rs3092856 | C | C/T | T | 0.0197 (T) | 1HZ | missense_variant | p.His1380Tyr |
| *ATM* | rs1800058 | C | C/T | T | 0.0092 (T) | 3HZ | missense_variant | p.Leu1420Phe |
| *ATM* | rs4988008 | C | C/T | T | 0.0014 (T) | 1HZ | synonymous_variant | p.Phe1491(p=) |
| *ATM* | rs1800889 | C | C/T | T | 0.0183 (T) | 6HZ | synonymous_variant | p.Pro1526(p=) |
| *ATM* | rs3092829 | T | T/C | C | 0.0179 (C) | 2HZ | intron_variant | IVS36-8T>C |
| *ATM* | rs1801516 | G | G/A | A | 0.0788 (A) | 11HZ | missense_variant | p.Asp1853Asn |
| *ATM* | rs3092910 | T | T/C | C | 0.0087 (C) | 1HZ | synonymous_variant | p.Ala1931(p=) |
| *ATM* | rs11212587 | G | G/A | A | 0.0014 (A) | 1HZ | missense_variant | p.Gly2023Arg |
| *ATM* | rs1800060 | G | G/A | A | 0.0046 (A) | 1HZ | missense_variant | p.Val2079Ile |
| *ATM* | rs56815840 | G | G/C | C | 0.0046 (C) | 1HZ | missense_variant | p.Ser2146Thr |
| *ATM* | rs759069006 | G | G/T | T | 0.000008 (T) | 1HZ | synonymous_variant | p.Leu2739(p=) |
| *IL10RA* | rs4252301 | G | G/C | C | 0.0027 (C) | 2HZ | synonymous_variant | p.Val7(p=) |
| *IL10RA* | rs75968822 | G | G/T | T | 0.0133 (T) | 1HZ | intron_variant | IVS1+224G>T |
| *IL10RA* | rs4252249 | G | A/A | A | 0.1145 (A) | 2HM/7HZ | synonymous_variant | p.Ala60(p=) |
| *IL10RA* | rs4252250 | C | C/G | G | 0.0087 (G) | 1HZ | missense_variant | p.Leu61Val |
| *IL10RA* | rs4252303 | G | G/A | A | 0.0037 (A) | 3HZ | missense_variant | p.Val113Ile |
| *IL10RA* | rs2256111 | A | G/G | G | 0.4757 (A) | 19HM/21HZ | synonymous_variant | p.Ala153(p=) |
| *IL10RA* | rs3135932 | A | A/G | G | 0.0824 (G) | 8HZ | missense_variant | p.Ser159Gly |
| *IL10RA* | rs2228054 | G | G/A | A | 0.1218 (A) | 1HM/3HZ | synonymous_variant | p.Pro175(p=) |
| *IL10RA* | rs2228055 | A | A/G | G | 0.1213 (G) | 1HM/2HZ | missense_variant | p.Ile224Val |
| *IL10RA* | rs2229113 | A | G/G | G | 0.1896 (A) | 31HM/11HZ | missense_variant | p.Arg351Gly |
| *IL10RA* | rs2229114 | C | C/T | T | 0.0284 (T) | 8HZ | missense_variant | p.Ser420Leu |
| *AICDA* | rs2028373 | G | G/A | A | 0.4826 (G) | 16HM/12HZ | synonymous_variant | p.His155(p=) |
| *AICDA* | rs2518144 | G | G/A | A | 0.4677 (G) | 9HM/25HZ | intron_variant | IVS2+16G>A |
| *IGHM* | rs368913002 | G | G/A | A | 0.0008 (A) | 1HZ | synonymous_variant | p.Pro358(p=) |
| *IGHM* | rs10287 | A | A/G | G | 0.0403 (G) | 1HZ | synonymous_variant | p.Thr281(p=) |
| *IGHM* | rs113517608 | G | G/A | A | 0.0256 (A) | 1HZ | synonymous_variant | p.Ile227(p=) |
| *IGHM* | rs12365 | C | A/A | A | 0.0673 (C) | 45HM/1HZ | missense_variant | p.Gly215Val |
| *IGHM* | rs1059216 | T | T/C | C | 0.2198 (C) | 5HZ | missense_variant | p.Ser191Gly |
| *IGHM* | rs1136534 | G | G/A | A | 0.4551 (G) | 14HM/19HZ | synonymous_variant | p.Ala107(p=) |
| *IGHM* | rs113762053 | G | G/C | C | 0.0394 (C) | 1HZ | missense_variant | p.Asp77Glu |
| *IGHM* | rs375883362 | G | G/A | A | 0.0004 (A) | 1HZ | synonymous_variant | p.Tyr65(p=) |
| *IGHM* | rs368782551 | C | C/T | T | 0.0013 (T) | 1HZ | missense_variant | p.Asp36Asn |
| *IL4R* | rs17548704 | C | C/T | T | 0.0060 (T) | 1HZ | synonymous_variant | p.Ser36(p=) |
| *IL4R* | rs1805010 | A | A/G | G | 0.4512 (G) | 8HM/20HZ | missense_variant | p.Ile75Val |
| *IL4R* | rs145184963 | C | C/T | T | 0.0055 (T) | 1HZ | synonymous_variant | p.Asn79(p=) |
| *IL4R* | rs112497527 | T | T/C | C | 0.0096 (C) | 1HZ | synonymous_variant | p.Asp97(p=) |
| *IL4R* | rs3024570 | G | G/A | A | 0.0394 (A) | 6HZ | intron_variant | IVS5-4G>A |
| *IL4R* | rs2234895 | C | C/T | T | 0.0476 (T) | 8HZ | synonymous_variant | p.Asn167(p=) |
| *IL4R* | rs3024638 | C | C/G | G | 0.0266 (G) | 3HZ | synonymous_variant | p.Thr256(p=) |
| *IL4R* | rs2234897 | T | T/C | C | 0.0137 (C) | 1HZ | synonymous_variant | p.Phe313(p=) |
| *IL4R* | rs1805011 | A | A/C | C | 0.2234 (C) | 1HM/10HZ | missense_variant | p.Glu400Ala |
| *IL4R* | rs2234898 | G | G/T | T | 0.2221 (T) | 1HM/10HZ | synonymous_variant | p.Leu414(p=) |
| *IL4R* | rs1805012 | T | T/C | C | 0.1016 (C) | 7HZ | missense_variant | p.Cys431Arg |
| *IL4R* | rs2234899 | C | C/T | T | 0.0343 (T) | 3HZ | synonymous_variant | p.Cys431(p=) |
| *IL4R* | rs2234900 | T | T/C | C | 0.2518 (C) | 2HM/10HZ | synonymous_variant | p.Leu433(p=) |
| *IL4R* | rs1805013 | C | C/T | T | 0.0252 (T) | 2HZ | missense_variant | p.Ser436Leu |
| *IL4R* | rs1805015 | T | T/C | C | 0.2097 (C) | 11HZ | missense_variant | p.Ser503Pro |
| *IL4R* | rs1801275 | A | A/G | G | 0.3452 (G) | 2HM/14HZ | missense_variant | p.Gln576Arg |
| *IL4R* | rs3024677 | G | G/A | A | 0.0293 (A) | 3HZ | missense_variant | p.Val579Ile |
| *IL4R* | rs3024678 | C | C/T | T | 0.0069 (T) | 1HZ | missense_variant | p.Pro675Ser |
| *IL4R* | rs1805016 | T | T/G | G | 0.1181 (G) | 1HM/5HZ | missense_variant | p.Ser752Ala |
| *IL4R* | rs1805014 | T | T/C | C | 0.0087 (C) | 2HZ | missense_variant | p.Ser786Pro |
| *IL4R* | rs2074570 | T | T/C | C | 0.0994 (C) | 2HZ | 3_prime_UTR_variant | 3'UTR*6T>C |
| *CD19* | rs142342927 | G | G/A | A | 0.0032 (A) | 2HZ | synonymous_variant | p.Ser127(p=) |
| *CD19* | rs2904880 | C | C/G | G | 0.1827 (C) | 25HM/14HZ | missense_variant | p.Leu174Val |
| *CD19* | rs148200569 | C | C/T | T | 0.0005 (T) | 1HZ | missense_variant | p.Pro176Leu |
| *CD19* | rs35979293 | G | G/T | T | 0.2624 (T) | 6HM/10HZ | synonymous_variant | p.Pro235(p=) |
| *CD19* | rs34763945 | G | G/A | A | 0.0256 (A) | 1HZ | missense_variant | p.Arg515His |
| *CD79B* | rs2070776 | A | G/G | G | 0.4006 (A) | 22HM/19HZ | synonymous_variant | p.Cys123(p=) |
| *CD23* | rs142442084 | A | A/G | G | 0.0004 (G) | 1HZ | missense_variant | p.Ser316Pro |
| *CD23* | rs8102872 | C | C/T | T | 0.0055 (T) | 2HZ | missense_variant | p.Arg284Gln |
| *CD23* | rs2228138 | G | A/A | A | 0.3622 (A) | 7HM/23HZ | synonymous_variant | p.His239(p=) |
| *CD23* | rs1042429 | C | C/T | T | 0.0032 (T) | 3HZ | synonymous_variant | p.Gly230(p=) |
| *CD23* | rs4996973 | A | A/G | G | 0.3622 (G) | 7HM/23HZ | intron_variant | IVS9-8T>C |
| *CD23* | rs28364072 | A | A/G | G | 0.3622 (G) | 7HM/23HZ | intron_variant | IVS9+7T>C |
| *CD23* | rs72558007 | C | C/T | T | 0.0160 (T) | 3HZ | missense_variant | p.Gly76Ser |
| *CD23* | rs2228137 | G | G/A | A | 0.1635 (A) | 3HM/12HZ | missense_variant | p.Arg62Trp |
| *CD23* | rs138917660 | C | C/T | T | 0.0014 (T) | 1HZ | missense_variant | p.Ala32Thr |
| *CD23* | rs2287866 | A | A/G | G | 0.1575 (G) | 1HM/5HZ | synonymous_variant | p.Arg20(p=) |
| *CD320* | rs2227289 | G | G/C | C | 0.1172 (C) | 1HM/3HZ | synonymous_variant | p.Thr279(p=) |
| *CD320* | rs2336573 | C | C/T | T | 0.1172 (T) | 1HM/3HZ | missense_variant | p.Gly220Arg |
| *CD320* | rs2232784 | G | G/A | A | 0.1168 (A) | 1HM/3HZ | synonymous_variant | p.Ser161(p=) |
| *CD320* | rs188158114 | C | C/T | T | 0.0005 (T) | 1HZ | missense_variant | p.Asp160Asn |
| *CD320* | rs2232783 | C | C/A | A | 0.1172 (A) | 1HM/3HZ | synonymous_variant | p.Thr149(p=) |
| *CD320* | rs2232776 | C | C/G | G | 0.1172 (G) | 1HM/3HZ | intron_variant | IVS1+10G>C |
| *CD320* | rs2232775 | T | T/C | C | 0.1470 (C) | 1HM/3HZ | missense_variant | p.Gln8Arg |
| *CD320* | rs2232773 | C | C/T | T | 0.1177 (T) | 1HM/3HZ | 5_prime_UTR_variant | 5'UTR-2G>A |
| *CD22* | rs146504496 | C | C/T | T | 0.0041 (T) | 1HZ | synonymous_variant | p.Cys265(p=) |
| *CD22* | rs145621000 | G | G/C | C | 0.0023 (C) | 5HZ | synonymous_variant | p.Leu325(p=) |
| *CD22* | rs61741013 | G | G/A | A | 0.0137 (A) | 1HZ | missense_variant | p.Ala391Thr |
| *CD22* | rs25677 | C | C/T | T | 0.0522 (T) | 3HZ | synonymous_variant | p.Cys484(p=) |
| *CD22* | rs35715143 | G | G/A | A | 0.0188 (A) | 1HZ | missense_variant | p.Gly551Arg |
| *CD22* | rs7259477 | G | G/A | A | 0.0027 (A) | 1HZ | synonymous_variant | p.Pro681(p=) |
| *CD22* | rs10406069 | G | G/A | A | 0.1442 (A) | 2HM/16HZ | missense_variant | p.Gly745Asp |
| *CD22* | rs79438722 | C | C/T | T | 0.0376 (T) | 2HZ | synonymous_variant | p.Cys790(p=) |
| *CD22* | rs117891073 | G | G/A | A | 0.0156 (A) | 1HZ | intron_variant | IVS13+10G>A |
| *TGF-β* | rs1800472 | G | G/A | A | 0.0137 (A) | 7HZ | missense_variant | p.Thr263Ile |
| *TGF-β* | rs55659002 | TG | TG/T | - | 0.0139 (-) | 4HZ | intron_variant | IVS4-8delC |
| *TGF-β* | rs202071274 | T | T/A | A | 0.0005 (A) | 1HZ | synonymous_variant | p.Thr137(p=) |
| *TGF-β* | rs1800471 | C | C/G | G | 0.0462 (G) | 5HZ | missense_variant | p.Arg25Pro |
| *TGF-β* | rs1800470 | G | A/A | A | 0.4446 (G) | 9HM/13HZ | missense_variant | p.Pro10Leu |
| *FCGRT* | rs150420714 | G | G/C | C | 0.0023 (C) | 2HZ | missense_variant | p.Lys132Asn |
| *FCGRT* | rs200294618 | G | G/A | A | 0.0005 (A) | 1HZ | missense_variant | p.Arg185His |
| *FCGRT* | rs2878342 | C | C/T | T | 0.1268 (T) | 1HM/5HZ | synonymous_variant | p.Arg194(p=) |
| *FCGRT* | rs3810194 | T | T/C | C | 0.1166 ( C) | 1HM/4HZ | intron_variant | IVS5+7T>C |
| *FCGRT* | rs768835093 | G | G/A | A | 0.00003 (A) | 1HZ | missense_variant | p.Ala365Thr |
| *FCAR* | rs1865096 | A | A/G | G | 0.2482 (A) | 32HM/11HZ | synonymous_variant | p.Arg108(p=) |
| *FCAR* | rs11666735 | G | G/A | A | 0.0627 (A) | 6HZ | missense_variant | p.Asp113Asn |
| *FCAR* | rs61735069 | T | T/C | C | 0.0545 (C) | 4HZ | synonymous_variant | p.Leu211(p=) |
| *FCAR* | rs79401710 | C | C/T | T | 0.0531 (T) | 4HZ | synonymous_variant | p.Pro224(p=) |
| *FCAR* | rs61735070 | G | G/A | A | 0.0019 (A) | 1HZ | synonymous_variant | p.Pro266(p=) |
| *FCAR* | rs16986050 | A | A/G | G | 0.1662 (G) | 3HM/11HZ | missense_variant | p.Ser269Gly |
| *ADA* | rs183914222 | G | G/A | A | 0.0005 (A) | 1HZ | 3_prime_UTR_variant | 3'UTR*4C>T |
| *ADA* | rs244076 | T | T/C | C | 0.2212 (C) | 4HM/11HZ | synonymous_variant | p.Val178(p=) |
| *ADA* | rs61737144 | C | C/T | T | 0.0298 (T) | 4HZ | synonymous_variant | p.Val130(p=) |
| *ADA* | rs11555566 | T | T/C | C | 0.0430 (C) | 5HZ | missense_variant | p.Lys80Arg |
| *ADA* | rs73598374 | C | C/T | T | 0.0440 (T) | 4HZ | missense_variant | p.Asp8Asn |
| *CD40* | rs1883832 | T | T/C | C | 0.2436 (T) | 21HM/18HZ | 5_prime_UTR_variant | 5'UTR-1T>C |
| *CD40* | rs11569321 | C | T/T | T | 0.0325 (T) | 1HM | missense_variant | p.Ser124Leu |
| *CD40* | rs7273698 | C | T/T | T | 0.0343 (T) | 1HM | synonymous_variant | p.Phe202(p=) |
| *CD40* | rs11086998 | C | C/G | G | 0.0211 (G) | 2HZ | missense_variant | p.Pro227Ala |
| *CHRNA4* | rs2229960 | A | G/G | G | 0.1204 (A) | 27HM | synonymous_variant | p.Cys409(p=) |
| *CHRNA4* | rs2229959 | C | A/A | A | 0.2321 (C) | 26HM/4HZ | synonymous_variant | p.Pro403(p=) |
| *CHRNA4* | rs781131183 | T | C/C | C | 0.00001 C | 1HM | missense_variant | p.Gln399Arg |
| *CHRNA4* | rs76270730 | G | G/A | A | 0.0005 (A) | 1HZ | synonymous_variant | p.Phe326(p=) |
| *CHRNA4* | rs1044394 | A | G/G | G | 0.1287 (A) | 41HM/4HZ | synonymous_variant | p.Cys226(p=) |
| *CHRNA4* | rs1044393 | A | G/G | G | 0.2147 (A) | 31HM/12HZ | synonymous_variant | p.Asp213(p=) |
| *CHRNA4* | rs2273506 | G | G/A | A | 0.0962 (A) | 2HM/10HZ | synonymous_variant | p.Leu63(p=) |
| *CHRNA4* | rs79739740 | C | T/T | T | 0.0229 (T) | 1HM/2HZ | synonymous_variant | p.Leu17(p=) |
| *CHRNA4* | rs6089898 | C | C/G | G | 0.0008 (G) | 1HZ | synonymous_variant | p.Ala8(p=) |
| *CHRNA4* | rs6090387 | G | G/C | C | 0.3997 (G) | 1HM/2HZ | 5_prime_UTR_variant | 5'UTR-30C>G |
| *IL10RB* | rs80027572 | G | G/A | A | 0.0023 (A) | 1HZ | missense_variant | p.Glu25Lys |
| *IL10RB* | rs2834167 | A | A/G | G | 0.3457 (G) | 1HM/16HZ | missense_variant | p.Lys47Glu |
| *VPREB1* | rs9619852 | G | G/A | A | 0.0554 (A) | 7HZ | synonymous_variant | p.Leu23(p=) |
| *VPREB1* | rs1320 | G | G/A | A | 0.0298 (A) | 1HZ | missense_variant | p.Asp76Asn |
| *VPREB1* | rs5995719 | C | T/T | T | 0.1493 (T) | 2HM/12HZ | synonymous_variant | p.Ser121(p=) |
| *VPREB1* | rs5995720 | G | A/A | A | 0.1511 (A) | 2HM/11HZ | missense_variant | p.Glu132Lys |
| *IGLL1* | rs8138122 | C | C/T | T | 0.0403 (T) | 2HZ | missense_variant | p.Arg189His |
| *IGLL1* | rs75088277 | G | G/A | A | 0.0343 (A) | 2HZ | synonymous_variant | p.Pro183(p=) |
| *IGLL1* | rs111903752 | A | A/T | T | 0.0449 (T) | 3HZ | missense_variant | p.Met162Lys |
| *IGLL1* | rs139571703 | C | C/T | T | 0.0353 (T) | 2HZ | missense_variant | p.Gly159Ser |
| *IGLL1* | rs145176864 | G | G/A | A | 0.0206 (A) | 2HZ | missense_variant | p.Pro155Leu |
| *IGLL1* | rs73157031 | G | G/A | A | 0.1346 (A) | 9HZ | missense_variant | p.Thr148Ile |
| *IGLL1* | rs114285337 | C | C/T | T | 0.0037 (T) | 1HZ | synonymous_variant | p.Gln103(p=) |
| *IGLL1* | rs115303391 | G | G/A | A | 0.0247 (A) | 1HZ | synonymous_variant | p.Ser100(p=) |
| *IGLL1* | rs116041505 | G | G/T | T | 0.0266 (T) | 3HZ | missense_variant | p.Thr95Lys |
| *IGLL1* | rs116275804 | G | G/A | A | 0.0229 (A) | 3HZ | synonymous_variant | p.Asn92(p=) |
| *IGLL1* | rs758781859 | G | G/A | A | 0.00004 (A) | 1HZ | missense_variant | p.Ser45Leu |
| *MIF* | rs189811391 | C | C/T | T | 0.0064 (T) | 1HZ | intron_variant | IVS2-9C>T |
| *MIF* | rs2070766 | C | C/G | G | 0.1900 (G) | 1HM/10HZ | intron_variant | IVS2-6C>G |
| *CD40LG* | rs1126535 | T | T/C | C | 0.1911 (C) | 4HM/2HZ | synonymous_variant | p.Leu50(p=) |
