# Supplementary material for: AGT haplotype in ITGA4 gene is related to antibody-mediated rejection in heart transplant patients
Source: PLoS One. 2019 Jul 23;14(7):e0219345. doi: 10.1371/journal.pone.0219345 (PMC6650139; doi:10.1371/journal.pone.0219345)
Supplement: S4 Table — (DOC) [file pone.0219345.s005.doc]

**S4 Table: Statistical analysis of the *ITGA4*** Haplotype association with response.

| ***ITGA4* Haplotype association with response (n=46,crude analysis)** | | | | | | | | | |
| --- | --- | --- | --- | --- | --- | --- | --- | --- | --- |
| **PROGRAM** |  | c.1845G>A | c.2633A>G | c.2883C>T | **Total Freq** | **Control Freq** | **AMR Freq** | **OR (95% CI)** | **P-value** |
| **SNPstats** | 1 | G | A | C | 0.6(55) | 0.8(35) | 0.4(20) | 1.0 | --- |
|  | **2** | **A** | **G** | **T** | **0.3(25)** | **0.1(6)** | **0.4(19)** | **11.0(2.3-53.6)** | **0.005** |
|  | 3 | A | A | T | 0.2(11) | 0.1(4) | 0.1(7) | 2.5(0.2-9.4) | 0.1 |
|  | 4 | G | A | T | 0.01(1) | 0.02(1) | NA | 1.0 | --- |
| **THESIAS** | 1 | G | A | C | 0.6(55) | 0.8(35) | 0.4(20) | 1.0 | --- |
|  | **2** | **A** | **G** | **T** | **0.3(25)** | **0.1(6)** | **0.4(19)** | **7.3(2.5-21.3)** | **<0.001** |
|  | 3 | A | A | T | 0.2(11) | 0.1(4) | 0.1(7) | 2.1(0.7-6.5) | 0.2 |
|  | 4 | G | A | T | 0.01(1) | 0.02(1) | 0.000(0) | 1.0 | --- |

NA:Not available.
